# Supplementary material for: Seedling recruitment under isolated trees in a tea plantation provides a template for forest restoration in eastern Africa
Source: PLoS One. 2021 May 6;16(5):e0250859. doi: 10.1371/journal.pone.0250859 (PMC8101954; doi:10.1371/journal.pone.0250859)
Supplement: S1 Table — Distances are shown to nearest forest with the source tree. Distances in parentheses are of source tree species located closest to the sampled recruit seedlings. (DOCX) [file pone.0250859.s001.docx]

**_________________________________________________________________________**

**Legacy tree species Legacy Minimum distance to (m)**

**tree code nearest source tree**
**_________________________________________________________________________**

**Exotic**

*Albizia* sp 2014_07 77 (78)^9^ (99)^9^(108)^9^
*Albizia odoratissima* 2018_13 55

*Grevillea robusta* 2014_01 63
*Grevillea robusta* 2014_02 31
*Grevillea robusta* 2014_03 27
*Grevillea robusta* 2014_04 77
*Grevillea robusta* 2014_05 164 (26)^1^
*Grevillea robusta* 2014_04 42

*Grevillea robusta* 2018_05 42

*Grevillea robusta* 2018_06 73

*Grevillea robusta* 2018_07 68

*Grevillea robusta* 2018_09 60

*Grevillea robusta* 2018_10 28 (32)^10^ (23)^12^

*Grevillea robusta* 2018_12 33

**Native**

*Anisophylea obtusifolia* 100 (3)^6^ (44)^6^
*Anthocleista grandflora* 40 (43)^7^ (68)^7^
*Ficus thonningi* 152
*Ficus sansibarica* 122 (37)^3^
*Parinari excelsa* 114
*Parinari excelsa* 111 (73)^4^
*Parinari excelsa* 99
*Parinari excelsa* 100 (34)^5^ (95)^5^
*Pouteria adolf-friedricii* 28 (1)^8^ (26)^8^ (39)^8^ (45)^8^

*Milicia excelsa*  41

*Milicia excelsa*  36

*Milicia excelsa*  28

*Milicia excelsa*  27 (27)^13^

*Milicia excelsa*  70 (63)^14^

*Milicia excelsa*  20 (20)^11^
**___________________________________________________________________**

^1^26 m to nearest source species *Rauvolfia* *caffra*

^2^ 50 m to nearest source species *Parinari excelsa*

^3^ 37 m to nearest source species *Parinari excelsa*

^4^ 73 m to nearest source species *Anisophyllea obtusifolia*

^5^ 34 m and 95 m to nearest source species of *Anthocleista* *grandiflora* and *Polyscias* *fulva*, respectively

^6^ 3 m and 44 m to nearest source species of *Parinari* *excelsa* and *Macaranga* *capensis*, respectively

^7^ 43 m and 68 m to nearest source species of *Parinari* *excelsa* and *Anisophyllea* *obtusifolia*, respectively

^8^ 1 m, 26 m, 39 m and 45 m to nearest source species of *Rauvolfia* *caffra*, *Parinari* *excelsa*, *Anthocleista* *grandiflora* and *Anisophyllea* *obtusifolia*, respectively

^9^ 78 m, 78 m, 79 m, and 108 m to nearest source species of *Maesopsis* *eminii* and *Shirakiopsis elliptica*, *Syncepalum* *cerasiferum* and *Parinari* *excelsa*, respectively

^10^ 32 m to nearest species *Bridelia* *micrantha*

^11^ 20 m to nearest species *Cedrela* *odorata*

^12^ 23 m to nearest species *Ficus* sp.

^13^ 27 m to nearest species *Ficus* sp.

^14^ 63 m to nearest species *Newtonia buchananni*
