# Supplementary material for: Seedling recruitment under isolated trees in a tea plantation provides a template for forest restoration in eastern Africa
Source: PLoS One. 2021 May 6;16(5):e0250859. doi: 10.1371/journal.pone.0250859 (PMC8101954; doi:10.1371/journal.pone.0250859)
Supplement: S2 Table — (DOCX) [file pone.0250859.s002.docx]

**Legacy Legacy Richness Abundance Richness Abundance Richness Abundance Richness Abundance**

**tree type Tree code species total total anim-disp anim-disp pioneer pioneer non-pioneer non-pioneer**

**Exotic** 2018_13 *Albizia* *odoratissima* 11 60 9 58 9 54 2 6

2014_07 *Albizia* sp 4 36 3 35 2 34 1 1

2014_01 *Grevillea* *robusta* 8 42 8 42 5 36 3 6

2014_02 *Grevillea* *robusta* 6 27 6 27 5 27 1 1

2014_03 *Grevillea* *robusta* 8 28 8 28 5 24 3 4

2014_04 *Grevillea* *robusta* 7 10 7 10 6 6 1 4

2014_05 *Grevillea* *robusta* 6 65 5 60 6 65 0 0

2018_04 *Grevillea* *robusta* 12 131 9 127 6 118 4 11

2018_05 *Grevillea* *robusta* 10 156 8 150 8 151 1 3

2018_06 *Grevillea* *robusta* 21 377 18 392 12 361 6 12

2018_07 *Grevillea* *robusta* 10 31 10 31 10 31 0 0

2018_09 *Grevillea* *robusta* 14 49 14 49 11 45 3 4

2018_10 *Grevillea* *robusta* 13 96 13 96 9 84 3 10

2018_12 *Grevillea* *robusta* 8 268 8 268 7 267 1 1

**Native** 2014_13 *Anisophylea* *obtusifolia* 5 65 5 65 3 53 2 12

2014_14 *Anthocleista* *grandflora* 8 22 8 22 5 17 3 5

2014_08 *Ficus* *sansibarica* 8 74 8 74 6 65 2 9

2014_09 *Ficus* *thonningi* 16 129 16 128 9 107 7 22

2018_01 *Milicia* *excelsa* 9 31 8 29 5 22 4 9

2018_02 *Milicia* *excelsa* 10 253 9 250 7 244 3 7

2018_03 *Milicia* *excelsa* 14 251 13 240 7 242 7 9

2018_08 *Milicia* *excelsa* 21 65 21 65 13 51 6 14

2018_11 *Milicia* *excelsa* 8 56 7 55 5 51 3 5

2018_14 *Milicia* *excelsa* 15 150 12 143 9 122 5 27

2014_06 *Parinari* *excelsa* 6 18 6 18 5 16 1 2

2014_10 *Parinari* *excelsa* 15 37 12 30 8 28 6 6

2014_11 *Parinari* *excelsa* 9 33 8 31 7 30 2 3

2014_12 *Parinari* *excelsa* 10 69 10 69 8 66 2 3

2014_15 *Pouteria* *adolf*-*friedricii* 12 171 11 170 8 165 3 5
